# Impairment of cyclopean surface processing by disparity-defined masking stimuli

**DOI:** 10.1167/jov.20.2.1

**Published:** 2020-02-10

**Authors:** Ross Goutcher, Paul B. Hibbard

**Affiliations:** Psychology Division, Faculty of Natural Sciences, University of Stirling, Stirling, UK; Department of Psychology, University of Essex, Colchester, UK

**Keywords:** 3D shape, cyclopean vision, disparity measurement, relative disparity

## Abstract

Binocular disparity signals allow for the estimation of three-dimensional shape, even in the absence of monocular depth cues. The perception of such disparity-defined form depends, however, on the linkage of multiple disparity measurements over space. Performance limitations in cyclopean tasks thus inform us about errors arising in disparity measurement and difficulties in the linkage of such measurements. We used a cyclopean orientation discrimination task to examine the perception of disparity-defined form. Participants were presented with random-dot sinusoidal modulations in depth and asked to report whether they were clockwise or counter-clockwise rotated. To assess the effect of different noise structures on measurement and linkage processes, task performance was measured in the presence of binocular, random-dot masks, structured as either antiphase depth sinusoids, or as random distributions of dots in depth. For a fixed number of surface dots, the ratio of mask-to-surface dots was varied to obtain thresholds for orientation discrimination. Antiphase masks were found to be more effective than random depth masks, requiring a lower mask-to-surface dot ratio to inhibit performance. For antiphase masks, performance improved with decreased cyclopean frequency, increased disparity amplitude, and/or an increase in the total number of stimulus dots. Although a cross-correlation model of disparity measurement could account for antiphase mask performance, random depth masking effects were consistent with limitations in relative disparity processing. This suggests that performance is noise-limited for antiphase masks and complexity-limited for random masks. We propose that use of differing mask types may prove effective in understanding these distinct forms of impairment.

## Introduction

The perception of three-dimensional (3D) form from binocular images depends on the successful encoding of binocular disparities. The measurement of these disparities begins at the first stages of visual cortex and continues through multiple cortical areas, with disparity selective cells found in V1, V2, hMT+, and elsewhere (Krug & Parker, 2011; Minini, Bridge & Parker, 2010; Parker, 2007; Uka & DeAngelis, 2003). Critically, the types of disparities to which cells are selective differs markedly across these areas.

At the earliest stages of cortical processing, disparity selectivity adheres to an absolute frame of reference, in which disparities are encoded in terms of differences in retinal locations (Cumming & Parker, 1999). Selectivity for absolute disparities has received significant attention over a prolonged period of years, with multiple variants of the disparity energy model having been used to account for aspects of both neural processing and behavioral performance (e.g., Goncalves & Welchman, 2017; Hibbard, Goutcher & Hunter, 2016; Read & Cumming, 2007). In particular, numerous researchers have considered whether limitations and/or biases in the perception of disparity-defined form may be accounted for through the action of absolute disparity selective mechanisms (e.g., Allenmark & Read, 2010; Allenmark & Read, 2011; Banks, Gepshtein & Landy, 2004, Filippini & Banks, 2009; Goutcher & Hibbard, 2014; Harris, 2014).

Beyond these early stages of processing, different response patterns are observed. Beginning at V2, and continuing through multiple cortical sites, neurons show selectivity for relative disparities, that is, for differences between absolute disparity measurements across space (Fang et al., 2018; Neri, Bridge & Heeger, 2004; Thomas, Cumming & Parker, 2002; Umeda, Tanabe & Fujita, 2007). Although sensitivity for relative disparities has not been modeled extensively (cf., Assee & Qian 2007; Hibbard & Goutcher, 2016; Zhaoping, 2002), numerous psychophysical observations suggest the importance of processes operating at this level (e.g., Deas & Wilcox, 2014; Deas & Wilcox, 2015; Glennerster & McKee, 1999; Glennerster & McKee, 2004; Goutcher & Hibbard, 2010; Goutcher, Connolly & Hibbard, 2018; Goutcher & Wilcox, 2016; Vreven, McKee & Verghese, 2002; Wardle & Gillam 2016). Such results indicate that the tuning properties of relative disparity mechanisms are critically important for understanding the perception of cyclopean form (Goutcher et al., 2018; Hibbard, 2005; Tyler, 1975; Tyler, 2012; Tyler & Kontsevich, 2001).

Given these distinct levels of processing for binocular disparity, there are multiple potential sources of limitation and impairment in the perception of cyclopean structure. Cyclopean perception (Julesz, 1971), our ability to perceive the 3D shape and structure of surfaces from binocular disparity alone, may, for example, be limited primarily by early absolute disparity measurement processes, as noted earlier. Alternatively, performance impairments may be more directly related to limitations in the measurement of relative disparities, or in the use of these measurements for the description of cyclopean form (e.g., Goutcher & Wilcox, 2016; Goutcher et al., 2018; Wardle & Gillam, 2016). In this article, we report the results of experiments measuring performance impairments for cyclopean surface perception in the presence of different forms of random-dot masking stimuli. Participants were asked to discriminate the orientation of a disparity-defined random-dot sinusoid. Masking stimuli differed in structure; one mask was a sinusoid in antiphase arrangement with the target, whereas the other mask contained dots randomly distributed in depth. Using these different masks allowed us to examine the extent to which limitations in the perception of cyclopean form could be accounted for by early absolute disparity measurement processes alone.

### Absolute disparity measurement

To understand how early measurement processes may be impaired, one must consider the nature of these processes and the signals on which they depend. A range of sources suggest that the early measurement of absolute disparities makes use of cross-correlation-like processes. This approach to disparity measurement has been supported by neurophysiological evidence (e.g., DeAngelis, Ohzawa & Freeman, 1991; Nienborg, Bridge, Parker & Cumming, 2004; Ohzawa, DeAngelis & Freeman, 1990) and biologically inspired computational modeling work (e.g., Chen & Qian, 2004; Fleet, Wagner & Heeger, 1996; Goncalvez & Welchman, 2017; Qian & Zhu, 1997; Read & Cumming, 2007) including some of the earliest algorithms designed for the purpose (Sperling, 1970). Cross-correlation-based algorithms have also been used to account for patterns of bias and sensitivity in human psychophysical performance (Allenmark & Read, 2010; Allenmark & Read, 2011; Banks, et al., 2004; Filippini & Banks, 2009; Goutcher & Hibbard, 2010; Goutcher & Hibbard, 2014; Harris, 2014).

**Figure 1.**
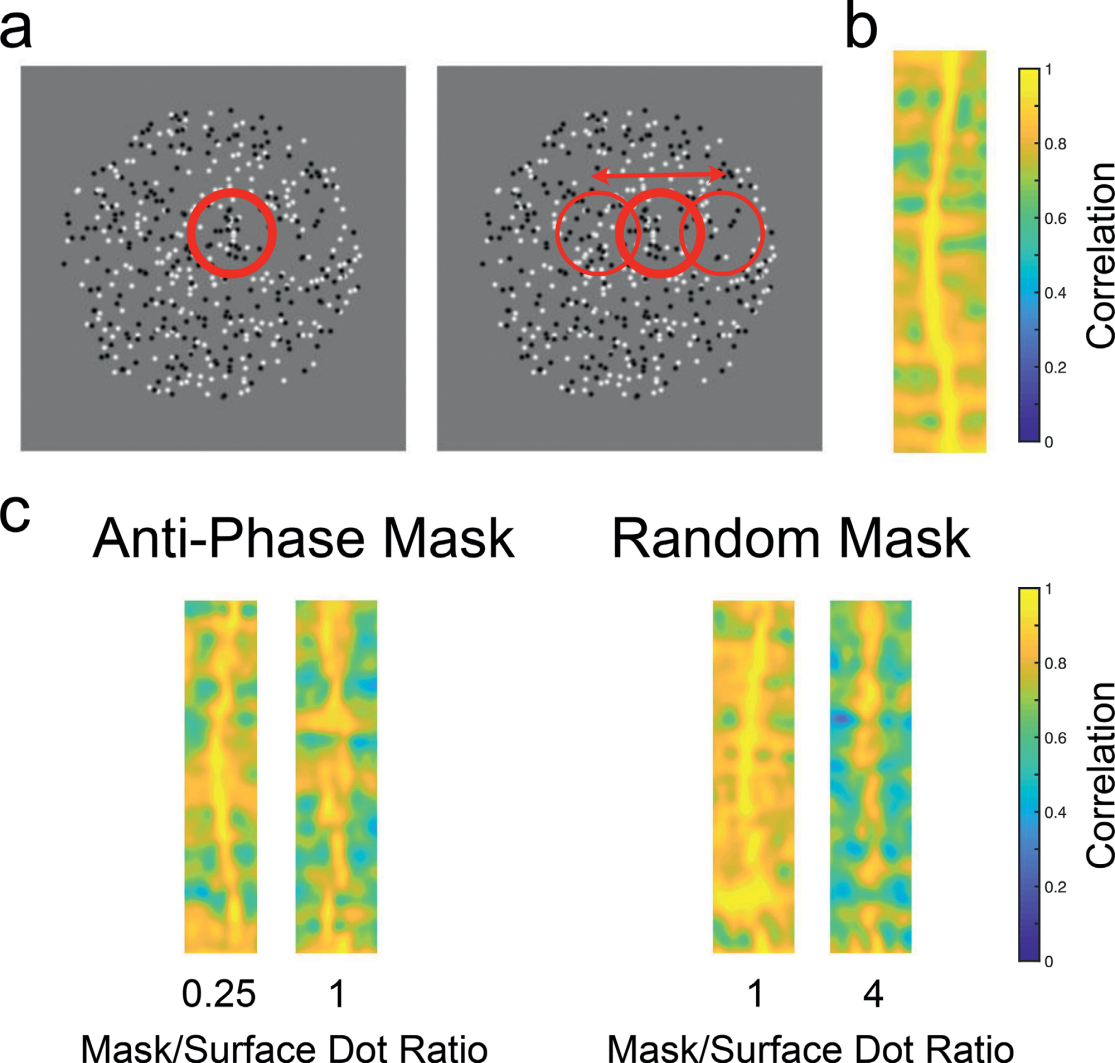
Illustration of absolute disparity measurement through cross-correlation and the effects of noise. (a) An example stereogram containing a sinusoidal modulation in depth, similar to the stimuli presented to participants in our experiments. Circles show local windowed patches to be compared by cross-correlation mechanisms. (b) y, disparity coordinate cross-section of the cross-correlation output for the example stimulus, using a smaller window standard deviation of 6.6 arcmin. The sinusoidal modulation is clearly visible. (c) Examples of the effects of antiphase and random disparity masking stimuli (see General methods for details). At low mask-to-surface dot ratios of the antiphase mask the wave form is still visible but is more difficult to discern at the limiting ratio of 1. For random disparity masks the waveform is still visible at this ratio but is more difficult to discern at much higher ratios. Note that the examples here have large disparity amplitudes (5.5 arcmin) and are for illustrative purposes only. They are not directly indicative of cross-correlation model performance.

Cross-correlation models for disparity measurement operate by comparing local image patches in one eye's image with image patches from the other eye's image (see Figure 1a,b). Disparity is defined by the positional offset between patches. The greater the correlation value at a given offset, the greater the signal for that disparity. Fundamentally, this means that such mechanisms act to detect local image structure. If patches are identical, save for their positional offset, then they will be strongly correlated. If, however, local structures in one eye's image are, for example, skewed relative to the other image, then correlation values will be lower. Cross-correlation mechanisms must therefore make a trade-off between the ability to measure local image structure and the likelihood that local structures will differ only in their positional offset. This trade-off depends on the size of window used for the cross-correlation calculation. Large correlation windows allow for greater sensitivity to local image structure but are also more likely to contain image areas with multiple disparities (Kanade & Okutomi, 1994).

The masking stimuli employed in this article affect local cross-correlation measurement in different ways. Masks in antiphase arrangement with the target depth sinusoid provided consistent local image structure at disparities other than the target disparity, increasing correlation at nontarget disparities while decreasing correlation at the target disparity (see Figure 1c). Moreover, disparities for the antiphase masking stimuli provide disparity information consistent with both possible stimulus orientations. Random disparity masks, however, provided signals that did not consistently favor one orientation or another. Random masks also produced random local changes in measured cross-correlation, rather than the consistent opposing signal seen in antiphase masks, leading to more general reductions in correlation, but only at higher levels of noise. This differing impact on cross-correlation calculations predicts that, for impairments at the level of early disparity measurement, random disparity masks will be far less effective than antiphase masks in disrupting cyclopean surface perception.

### Descriptions of relative disparities

Although cross-correlation mechanisms are limited by their sensitivity to disruptions of local image structure, cyclopean surface perception may also be impaired at the level of relative disparity processing. Such relative disparity impairment may take the form of noise in the measurement of relative disparity itself or may be related to the visual system's description of available relative disparity signals. Thus one may distinguish between noise-limited performance, in which basic measurements are themselves impaired, and complexity-limited performance, in which cyclopean structure is obscured through erroneous links between target relevant and irrelevant information. This idea of complexity-limited performance is commonly encountered in research focused on perceptual grouping (e.g., Elder & Goldberg, 2002; Field, Hayes & Hess, 1993; Watt, Ledgeway & Dakin, 2008), in which a classic example of complexity-limited performance is found in contour detection tasks. Here the ability to detect a target contour among distractor elements is impaired by complexity-related factors, such as spurious groupings of distractor elements, or the disruption of grouping mechanisms for target elements due to intervening distractors (Watt et al., 2008).

In this article, we provide a *minimal linkage* model of the relative disparity content of our cyclopean masking stimuli, describing the 3D spatial relationships between pairs of stimulus elements. This model is aimed at understanding complexity-based limitations in cyclopean surface perception, which arise owing to the relative disparity signals provided by the masking stimulus itself and through its grouping with the target surface. It should not be considered as a model of cyclopean processing per se, but as a description of stimulus content. As such, this linking model does not consider possible sources of measurement noise and is concerned only with the extent to which simple descriptions of relative disparity content may be used to discriminate cyclopean form. Our model describes relative disparity content in terms of the orientation, elevation, and length of the sets of dipoles connecting pairs of stimulus dots. By making explicit the task-limiting effects of such 3D spatial relationships, this model shows how different forms of noise may disrupt processing at the cyclopean level.

The discrimination of cyclopean orientation in this dipole model depends on the orientation (i.e., the angle in the x, y plane) and elevation (i.e., angle in the x, disparity plane) statistics of dipole distributions. For a noise-free disparity-defined sinusoid, the spread of dipole elevations will be smallest at the target orientation and largest at the orthogonal orientation. Performance limitations for this model therefore depend on the statistics of mask-related dipoles (i.e., any dipole in which one or both points are defined by a mask element) and the extent to which they interfere with expected distributions. If mask-related dipoles add noise to orientation and elevation distributions, then they will impair performance.

This argument leads to an interesting prediction: although antiphase masks will add noise to dipole distributions through increased zero elevation dipoles at nontarget orientations (i.e., for dipoles linking target and mask dots with equivalent absolute disparities), they will also add further zero elevation dipoles at the target orientation (i.e., for dipoles linking pairs of same disparity mask dots). The random disparity mask would, conversely, add noise to dipole distributions indiscriminately. As such, contrary to the predictions of noise-limited cross-correlation models, complexity-limited orientation discrimination performance should be less affected by an antiphase mask than by a random disparity mask. We test this possibility in this article, comparing human performance with random disparity and antiphase masks to the performance of both cross-correlation and dipole models of cyclopean orientation discrimination. Our results show that, although patterns of performance limitations are consistent with a role for early measurement noise, the effects of random disparity masks also demonstrate the key role played by complexity-limited grouping processes in cyclopean processing. These results provide a first step beyond the initial disparity matching processes in our understanding of cyclopean surface perception.

## General methods

### Participants

Five observers, including author RG, participated in a total of four experiments. Of these five observers, three participated in Experiment 1, with four participating in Experiments 2 and 3. All five observers took part in Experiment 4. All were experienced psychophysical observers with normal, or corrected-to-normal vision, and stereoacuity of at least 40 seconds of arc, as measured by the Random Dot 2 Stereo Acuity Test (Vision Assessment Corp., Elk Grove Village, IL). All experimental procedures were approved by the University of Stirling Psychology ethics board, in accordance with the guidelines of the British Psychological Society and the Declaration of Helsinki.

### Stimuli

**Figure 2.**
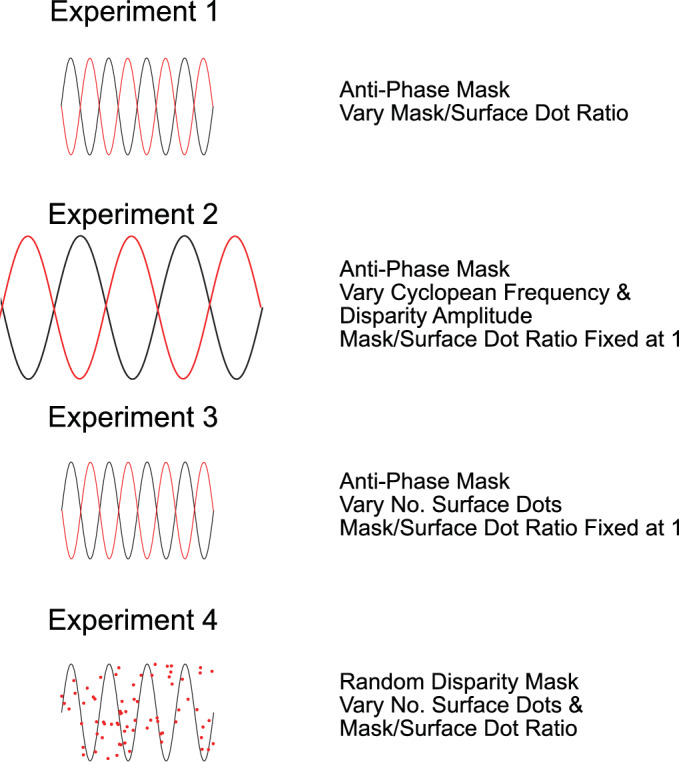
Summary of the stimulus manipulations used across all four experiments. Target surfaces are illustrated as black sinusoidal curves, while illustrations of mask stimuli are shown in red. Stimuli in the experiments were disparity-defined random-dot sinusoidal surfaces, oriented ±20˚ from vertical.

In each experiment, participants were presented with a random-dot stereogram depicting a sinusoidal modulation in depth. Gratings were oriented at ±20˚, with the sign of orientation chosen, at random, at the beginning of each trial. Except in Experiment 3, gratings were always of amplitude 1.1 arcmin and cyclopean frequency 0.84 cpd, with phase kept constant across all stimuli. The elements comprising the stimulus were circular dots of diameter 5.74 arcmin. Dots could be either black or white and were randomly distributed within a circular window of diameter 4.98˚. The number of dots used to define the sinusoid varied between 60 and 700 across experiments, equivalent to target surface dot densities of between 3.1 and 35.9 dots per degree^2^, respectively. In addition to dots defining the depth sinusoid, each experiment included masking dot stimuli. These were structured as either a random disparity distribution, or as a phase-shifted depth sinusoid of identical orientation, amplitude, and frequency as the target surface. Conditions for each experiment are described in full detail throughout the article and are summarized in Figure 2.

#### Design and procedure

Stimulus presentation and data recording were carried out using a MacPro computer with a 49 × 31 cm Apple Cinema HD Display (Apple Inc., Cupertino, CA). Display resolution was 1920 × 1200 pixels, with a refresh rate of 60Hz. Each pixel measured 1.1 arcmin, at the 76.4 cm viewing distance. Stimuli were generated using MATLAB (Mathworks Inc., Natick, MA) together with the Psychophysics Toolbox extensions (Brainard, 1997; Pelli, 1997; Kleiner, Brainard & Pelli, 2007). Display luminance ranged between 0.18 cdm^−2^ and 45.7 cdm^−2^. The display was calibrated using a SpyderPro2 calibration device (DataColor, Dietlikon, Switzerland) to ensure a linear luminance output. The presentation of binocular images was achieved using a mirror stereoscope, calibrated to ensure that vergence and accommodation were consistent with the stimulus viewing distance. All experiments were conducted in a darkened laboratory, with head movements restricted using a HeadSpot chinrest (UCHO, Houston, TX).

In each experiment, participants were presented with a single random-dot stereogram depicting a sinusoidal modulation in depth and asked to judge whether it was oriented in a clockwise or counter-clockwise direction. Stimuli were presented for 250 ms and were preceded by the 500 ms presentation of a fixation cross. Participants responded by pressing one of two keys on a keyboard. Each key press initiated the presentation of the next trial. Participants viewed the stimuli in random order, over multiple blocks, resulting in a minimum of 30 repeated trials per condition in each experiment. Stimulus presentation and manner of response were identical across all experiments.

## Modeling cyclopean orientation discrimination

To understand the factors limiting orientation discrimination performance, we devised two models of cyclopean orientation discrimination. One of these models, the cross-correlation model, targeted the impact of measurement noise at the level of absolute disparities. The second model, the dipole model, examined the impact of grouping processes on cyclopean orientation discrimination. Thus these models selectively assessed the contributions of noise-limited factors in disparity measurement and complexity-limited descriptions of relative disparity structure for cyclopean orientation perception. We detail these models later.

### Cross-correlation models for disparity estimation

To assess performance in our psychophysical tasks in terms of limitations in early disparity measurement processes, we employed a normalized cross-correlation model similar to those used by Filippini and Banks (2009) and Allenmark and Read (2010, 2011). For each experiment, the cross-correlator passed a Gaussian windowed patch from one eye's image over the other image at a range of different horizontal offsets, equivalent to a range of horizontal disparities. The correlation at each disparity was calculated according to Equation 1,

(1) $$\begin{array}{c} \;\begin{array}{c} c\left(\delta_{x}\right)=\frac{\sum_{\left(x,y\in W_{L}\right)}\;\left[\left(L_{\left(x,y\right)}\;-\;\mu_{L}\right)\;\left(R_{\left(x-\delta_{x},y\right)}\;-\;\mu_{R}\right)\right]}{\sqrt{\sum_{\left(x,y\in W_{L}\right)}{\left(L_{\left(x,y\right)}-\mu_{L}\right)}^{2}}\sqrt{\sum_{\left(x,y\in W_{L}\right)}{\left(R_{\left(x-\delta_{x},y\right)}-\mu_{R}\right)}^{2}}} \end{array} \end{array}$$

where δ_x_ is the disparity given by the cross-correlation offset, *w_L_* is the local windowed patch in the left image, *L* and *R* are left and right images, and μ_L_ and μ_R_ are the mean luminance values for left and right window regions.

**Figure 3.**
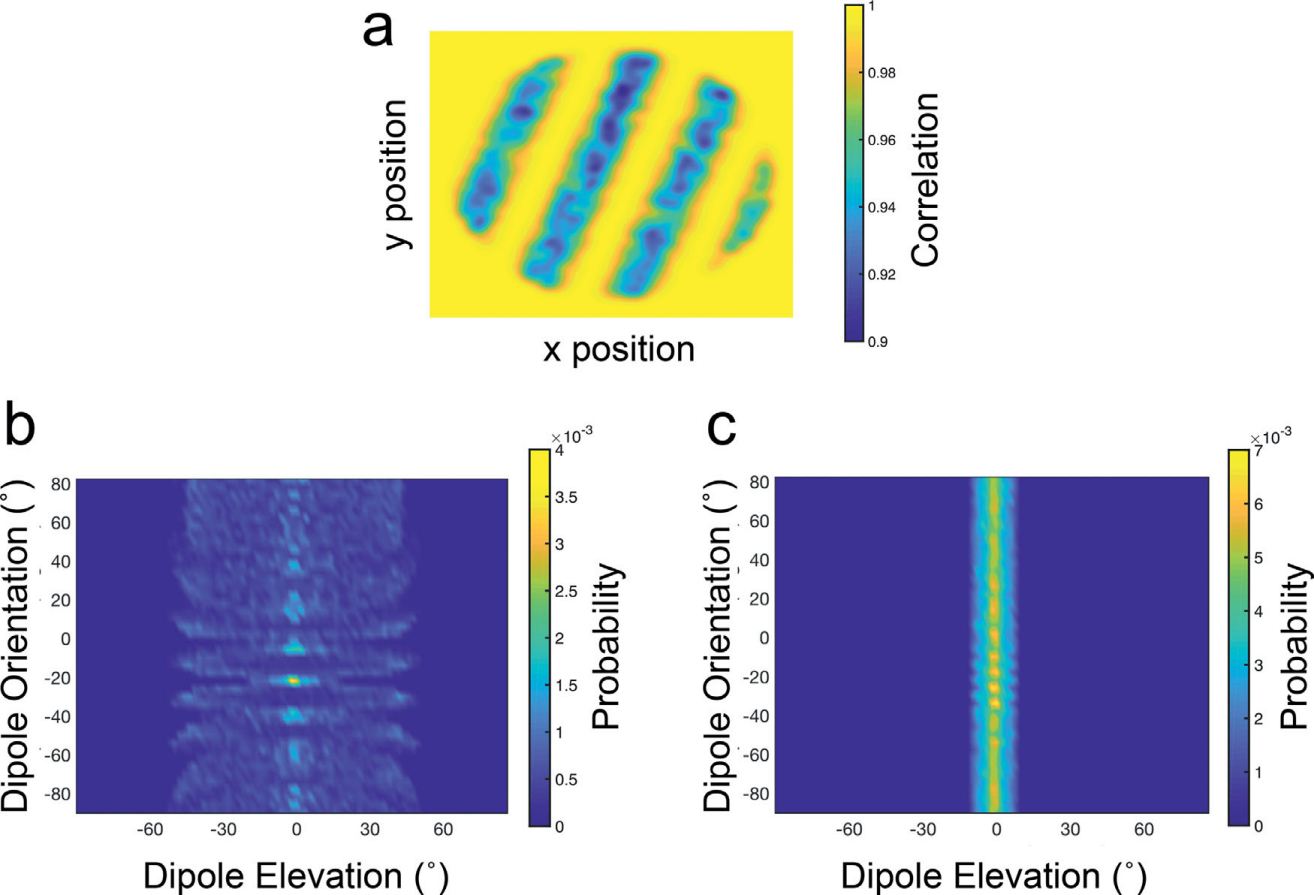
Example templates from (a) the cross-correlation model and (b,c) the dipole model. (a) An example template for a clockwise oriented depth sinusoid at a disparity of 1.1 arcmin. The template shows a “no mask” condition, for a window standard deviation of 17.6 arcmin. (b) An example template for the dipole model, showing the joint probability of dipole elevation and orientation, for dipole lengths of between 14 and 22 arcmin. (c) An example dipole template for longer dipoles of between 163 and 172 arcmin.

Using this model, we measured the correlation of left and right eye images across a range of disparities for Gaussian windowed patches centered on each point of the stimulus image. Cross-correlation calculations were always performed with the same window size, regardless of disparity. The resulting correlation maps provide evidence of the disparity structure of each stimulus (see Figure 3a). Performance in psychophysical tasks requires a further decision-making stage, however. For our model to make a decision about the orientation of each stimulus, we adopted a template matching approach similar to that used by Allenmark and Read (2010, 2011). Using this approach, cross-correlation outputs for a stimulus are compared with a family of templates that provide the typical cross-correlation response for a range of task relevant stimuli. Templates were generated for each experiment by obtaining cross-correlation maps for 50 repeated trials of each stimulus level, at both possible stimulus orientations. Templates were generated separately for each experiment, and no templates were shared between experiments. During testing, templates were correlated with the cross-correlation map generated for each trial. Orientation judgements were made on each trial by selecting the highest template-stimulus correlation, under a winner-takes-all rule. Note that at no stage does this template matching approach make an estimate of the absolute disparity of any region of the stimulus. Instead, model responses depend on variation in cross-correlation measures across x, y, disparity space.

To assess the extent to which cyclopean orientation judgements could be impaired at the level of absolute disparity measurement, we varied the size of the Gaussian windowed patches used by our cross-correlation model. For Experiments 1, 3, and 4, in which the amplitude and cyclopean frequency of the depth sinusoid were held constant at 1.1 arcmin and 0.84 cpd, respectively, cross-correlation windows were defined as two-dimensional Gaussians with standard deviations of between 3.3 and 35.2 arcmin. Window standard deviations in Experiment 2 varied between 2.2 and 45.1 arcmin. These sizes were selected to take into account the manipulations of disparity amplitude and cyclopean frequency made in this experiment. Window sizes were selected following the relationship laid out in Allenmark and Read (2011) in which a cross-correlation window's standard deviation is defined relative to its absolute disparity tuning as

(2) σ=3+0.27δ

where σ is the standard deviation of the cross-correlation window and δ is its disparity tuning. We also considered the relationship between cyclopean frequency and correlation window size, using the formula proposed by Nienborg et al (2004), in which the relationship between window standard deviation and cyclopean frequency tuning is given as

(3) $$\sigma =1/\left(2\pi \xi \right)$$

where ξ is the cutoff frequency above which disparity modulations are no longer detectable by windows of standard deviation σ. Although these equations guided the choice of window standard deviation across models, they were not used to covary window size and cross-correlation offset (i.e., disparity) as in Allenmark and Read (2011). Instead, window-size was held constant across disparities. This means that model performance reflects the ability of a single correlation window size to measure absolute disparity across the stimulus. Modeling was conducted in this fashion to consider the role of window size only, rather than more complex, physiologically, and behaviorally derived, concepts.

### A dipole model for cyclopean orientation discrimination

Although cross-correlation approaches to the modeling of disparity measurement can account for early noise factors, such as correspondence and measurement noise (e.g., Goutcher & Hibbard, 2014), they are not well-suited to addressing issues of cyclopean structure and do little to account for complexity-based processing limitations. In addition, given that the measurements of disparity obtained from cross-correlation procedures are in absolute coordinates, these models offer no obvious means by which to compare the structure of stimuli at different absolute depths, or at different slants. Instead, such comparisons require measurements based on differences in disparity. Sensitivity to such relative disparities has been noted in multiple areas of cortex (Fang et al., 2018; Neri et al., 2004; Parker, 2007; Thomas et al., 2002; Umeda et al., 2007).

Here we consider an approach to describing disparity differences independently of absolute disparity measurement. The starting point for this approach is to assume that the visual system possesses error-free measurements of the absolute disparity of each stimulus dot. In other words, rather than beginning with the stereoscopic image pair, this model assumes that the coordinates of each point in x, y, disparity space are known. Although this is an unrealistic assumption (cf., Stevenson, Cormack & Schor, 1989; Wardle, Bex, Cass & Alais, 2012), it ensures that any observed performance limitations arise from uncertainty concerning the description of relative disparities, rather than uncertainty about their measurement. Note that, even aside from the assumption of error-free measures of absolute disparity, this model should not be considered as an attempt to map out the processes involved in the measurement and description of relative disparities. Instead, this model should be understood as a description of the relative disparity content of our stimuli and of the efficacy of this content for the discrimination of cyclopean orientation. It is a description of stimulus information, not a model of disparity processing.

Our model generated descriptions of disparity differences by calculating the orientation, elevation, and length of sets of dipoles linking each and every pair of dots in a stimulus. These dipoles capture the simplest 3D structural information about the relationship between pairs of points on the surface. On the basis of this element for describing relative disparity, we refer to this model as the *dipole model*. For each dipole, orientation (*O,* angle in the x, y plane), length (*L*), and elevation from the image plane (*E,* angle in the x, disparity plane) were calculated using the following equations:

(4) $$O=\tan^{-1}\frac{y_{2}-y_{1}}{x_{2}-x_{1}}$$

(5) $$L=\sqrt{{\left(x_{2}-x_{1}\right)}^{2}+{\left(y_{2}-y_{1}\right)}^{2}}$$

(6) $$E=\tan^{-1}\left[\left(z_{2}-z_{1}\right)/L\right]$$

where x and y values define the coordinates of element positions in terms of their cyclopean direction, and z values represent an element's absolute disparity.

These measurements were used to define the joint probability distribution *p(E, O, L)* for each stimulus. Note that the measurement of these, and only these, properties means that the dipole model has no access to the absolute disparity information used as its input. Task performance with the dipole model therefore depends on only the most fundamental description of relative disparity information available in the stimulus. This also means that task performance for the dipole model depends on the efficacy of this relative disparity information as a description of the cyclopean orientation of the stimulus. Model performance thus reflects a complexity-based limitation in the model's summary description of the stimulus, not its measurement of either absolute or relative disparity content. The limitations revealed by our analysis thus reflect the relative depth information available to cyclopean processes, rather than any constraints imposed by the mechanisms themselves.

As with our cross-correlation model, we used a template-matching approach to allow for orientation discrimination judgements. Template dipole distributions were generated for 50 repeated trials of each stimulus level and orientation, across all four experiments (see Figure 3b,c). For each experiment, these templates were correlated with the stimulus dipole distributions generated on each trial, with the selected orientation determined by a winner-takes-all decision rule. As with the cross-correlation model, the generation of templates was independent between experiments.

Examination of the template in Figure 3b (for dipoles with lengths of between 14 and 22 arcmin) shows the relative disparity structures available in the dipole distributions. The distribution of dipoles peaks for elevations of zero at the target orientation of –20˚. Note here that variability in elevation is far lower at this target orientation. Conversely, elevation variability is high at the orthogonal orientation of 70˚. Such structure is not so visible for longer dipoles of between 163 and 172 arcmin (Figure 3c) because such dipoles are longer than the wavelength of the depth sinusoid. The range of lengths over which dipole distributions provide structured information about the cyclopean orientation of the target surface will depend on both the disparity amplitude and cyclopean frequency of the stimulus.

## Experiment 1: Antiphase masking of cyclopean orientation

### Stimuli

In Experiment 1, participants were presented with random-dot depth sinusoids in the presence of a cyclopean masking stimulus. The mask was structured as a random-dot depth sinusoid, with identical orientation, amplitude, and frequency as the target surface, but with opposite phase. Dot density for the target sinusoid was kept at 6.2 dots per degree^2^ throughout the experiment. This meant that the target surface was defined by 120 randomly distributed dots. Mask-to-surface dot ratios were varied uniformly across seven levels, from a ratio of 0.2 to a ratio of 1 to obtain thresholds for orientation discrimination. Note that for antiphase masks, 1 was the maximum possible mask-to-surface dot ratio. Values >1 indicate conditions in which the antiphase mask surface becomes the *de facto* target.

### Results and discussion

#### Psychophysical results

**Figure 4.**
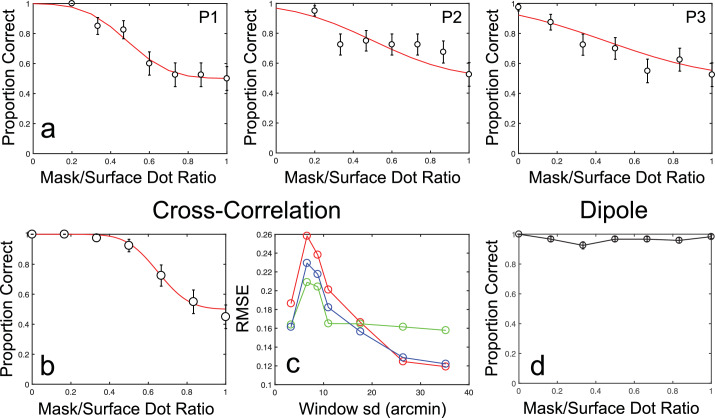
Results from Experiment 1. (a) Results for individual participants (P1 is author RG), together with fitted scaled cumulative Gaussian functions. Error bars show binomial standard errors. (b) Example results from the cross-correlation model, at a window standard deviation of 17.6 arcmin. (c) Cross-correlation model prediction error shown as RMSE against window standard deviation. Each color shows the error for an individual participant. (d) Dipole model performance as a function of mask-to-surface dot ratio.

Proportion correct orientation discrimination scores were calculated for each mask-to-surface dot ratio and fit with a decreasing scaled cumulative Gaussian function. Seventy-five percent correct thresholds were obtained from these fitted functions for each participant as a measure of the masking effect of the antiphase dots, with larger thresholds indicating an increased tolerance for masking noise. These results are shown in Figure 4a. As is evident from this figure, increasing the mask-to-surface dot ratio decreased orientation discrimination performance, with participants reaching chance performance before the limiting ratio of 1. Thresholds for 75% correct performance were at ratios of 0.45, 0.49, and 0.50 for the three observers, with 95% confidence intervals (CIs) of ±0.004, ± 0.025, and ±0.08, respectively.

#### Modeling results

Figures 4b and 4c show the performance of the cross-correlation model when presented with the antiphase mask stimuli. As with human observers, the performance of the cross-correlation model decreased with increasing mask-to-surface dot ratio. Increasing the window size led to a reduction in thresholds. Near-ceiling performance was observed for the smallest window sizes, of standard deviation 3.3 and 6.6 arcmin, with proportion correct scores remaining above chance level, even at the maximum mask-to-surface dot ratio. At larger window sizes, performance more closely approximated that of human observers. At the very largest window sizes, however, more generalized performance impairments were observed at all mask-to-surface dot ratios (i.e., performance was at or below threshold for all mask-to-surface dot ratios). We quantified the effects of window sizes by running bootstrapped simulations to estimate the range of root mean squared errors (RMSE) across each tested window standard deviation. For each of 5000 simulations we found the window standard deviation that provided the smallest RMSE. From this distribution of minimum RMSEs we found the best-fitting window standard deviation, using maximum likelihood estimation. The results of this analysis show that discrepancies between human and model performance were, on average, lowest for window sizes of around 24.5 arcmin. The 95% CIs for the distribution of minimum RMSEs were rather large, however (±20.5 arcmin). Although this is largely a factor of the comparatively small impact of increases in window standard deviation beyond 11 arcmin (see Figure 4c), it suggests, beyond smaller window sizes in which manipulations of mask-to-surface dot ratio have little effect, there are a wide range of window sizes consistent with human-like performance.

In contrast to the best-fitting cross-correlation model, the dipole model shows no performance impairments when presented with antiphase masking stimuli (Figure 4d). These results are consistent with the antiphase masking stimulus primarily limiting performance through its effect on the initial measurement of absolute binocular disparity, rather than through the resulting relations between points. The antiphase mask therefore appears to impose noise-based limitations on performance. Critically, however, such effects require a reliance on measurement processes operating at very coarse scales. The equations proposed by Allenmark and Read (2011) and Nienborg et al (2004) for the relationship between correlation window size and, respectively, the parameters of absolute disparity and cyclopean frequency, suggest that our experimental stimulus is processed by units with window standard deviations of between 3.3 and 11 arcmin. In comparison, cross-correlation model performance at these standard deviations exceeds that of human observers by some margin. We further examine these issues in Experiment 2 through manipulation of cyclopean frequency and disparity amplitude for stimuli at the limiting 1:1 mask-to-surface dot ratio. Both the frequency and amplitude are expected to influence the size of the correlation window that is relevant for the perception of depth. Higher spatial frequencies should rely on smaller correlation windows to capture finer spatial structure (Banks et al., 2004; Nienborg et al., 2004). Disparity magnitude is also related to window size, with the processing of larger magnitudes of disparity associated with larger correlation windows (Allenmark & Read, 2011; Prince, Cumming & Parker, 2002; Smallman & MacLeod, 1994).

## Experiment 2: Measuring effects of cyclopean frequency and disparity amplitude

### Stimuli

In Experiment 2, participants were once again presented with depth sinusoids in the presence of an antiphase mask. Here, however, the ratio of mask-to-surface dots was kept constant, at the maximum value of 1. Thus rather than measuring the ratio of mask-to-surface dots required to reduce performance to threshold level, Experiment 2 examined whether changes in the structure of the depth sinusoid could improve task performance. To this end, we varied the amplitude and cyclopean frequency of both surface and mask sinusoids. Cyclopean frequency was either 0.21, 0.42, 0.63, or 0.84 cpd, while amplitude was varied uniformly across five levels between 1.1 and 2.75 arcmin. In all cases, the frequency and amplitude of the target and mask were identical and differed only in their phase.

### Results and discussion

#### Psychophysical results

**Figure 5.**
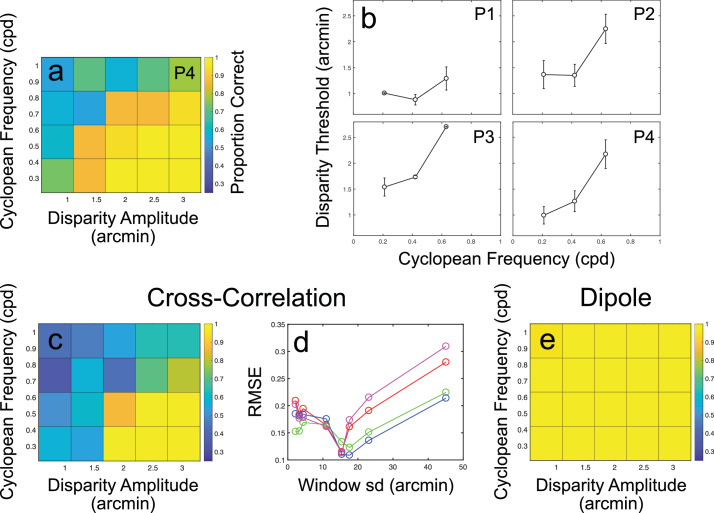
Psychophysical and modeling results for Experiment 2. (a) Results for an example participant (P4) showing increases in proportion correct scores with decreasing cyclopean frequency and increasing disparity amplitude. (b) Results for each participant (P1 is author RG) shown as 75% correct disparity amplitude thresholds across each level of cyclopean frequency. Error bars show the standard deviation of best-fitting thresholds, obtained via bootstrapped resampling. (c) Results for the cross-correlation model with a window standard deviation of 17.6 arcmin (close to the best-fitting window size; see Experiment 2 Results and discussion for details). (d) Prediction error for the cross-correlation model, shown as RMSE against window standard deviation. Each color shows the errors for a different participant. (e) Results for the dipole model, showing ceiling-level performance at all cyclopean frequencies and disparity amplitudes.

Figures 5a and 5b show the results for Experiment 2, in which cyclopean frequency and amplitude were manipulated, given a constant mask-to-surface dot ratio at the limiting value of 1. At this value, performance was again at chance level for stimuli with a frequency of 0.84 cpd and an amplitude of 1.1 arcmin. Combined decreases in cyclopean frequency and increases in amplitude led to improvements in orientation discrimination performance. These improvements are plotted for each participant in Figure 5b as 75% correct thresholds for disparity amplitude at each cyclopean frequency. Note that such thresholds could not be found for the highest tested frequency of 0.84 cpd. Thresholds increased with increasing cyclopean frequency, from an average disparity amplitude of 1.12 arcmin at the 0.21 cpd frequency, to 1.92 arcmin at a frequency of 0.63 cpd (*F*_2,6_ = 21.03, *p* = 0.0019 on a Repeated Measures analysis of variance [ANOVA]). Thresholds for the 0.84 cpd condition exceeded the tested disparity amplitude range. Across observers, disparity threshold 95% CIs ranged from ±0.02 to ±0.49 arcmin for the 0.21 cpd frequency, ±0.05 to ±0.38 arcmin for the 0.42 cpd frequency, and ±0.01 to ±0.5 arcmin for the 0.63 cpd frequency.

#### Modeling results

As in Experiment 1, the cross-correlation model provided a close match to human performance for a subset of window sizes. Model performance is shown for an example window size and as RMSE across window sizes in Figures 5c and 5d. As with mask-to-surface dot ratio manipulations in Experiment 1, the dipole model shows little to no effect for manipulations of cyclopean frequency and amplitude, maintaining ceiling-level performance (Figure 5e).

These results are again consistent with a primarily noise-based effect of the antiphase mask at the level of absolute disparity measurement. In contrast with Experiment 1, however, the cross-correlation model performs most similarly to human observers with intermediate window sizes. Best-fitting window standard deviations for Experiment 2, calculated using the same approach as for Experiment 1, were approximately 15.5 arcmin, with 95% CIs of ±6.74 arcmin (Figure 5d). Given that, for most conditions, disparities are larger and cyclopean frequencies lower than in Experiment 1, one should, if anything, expect the best-fitting window size to increase. That this is not the case suggests that impairment of absolute disparity measurements is not the sole factor affecting cyclopean surface perception in this task. The limiting effect of window size does, however, suggest that performance impairments are due to some form of measurement process, especially given the ceiling-level performance of the dipole model. We consider potential measurement-related factors in the Discussion.

## Experiment 3: Measuring the effects of element number

### Stimuli

As in Experiment 2, Experiment 3 sought to examine the effect of changes on surface structure on orientation discrimination performance. Participants in Experiment 3 were therefore presented with a series of stimuli in which the mask-to-surface dot ratio was again kept constant at the maximum value of 1. In this experiment, rather than varying the structure of the depth sinusoid, we varied the total number of stimulus elements. Increasing the number of dots was expected to improve performance, despite the maintenance of the 1:1 mask-to-surface dot ratio, as increasing the number of stimulus elements improves the sampling of the surface structure. Dot numbers were varied uniformly across seven levels, from 100 to 700 target dots. The total number of dots in each stimulus thus varied from 200 to 1400, equivalent to total dot densities of between 10.3 and 71.9 dots per degree^2^, and target surface densities of between 5.1 and 35.9 dots per degree^2^.

### Results and discussion

#### Psychophysical results

**Figure 6.**
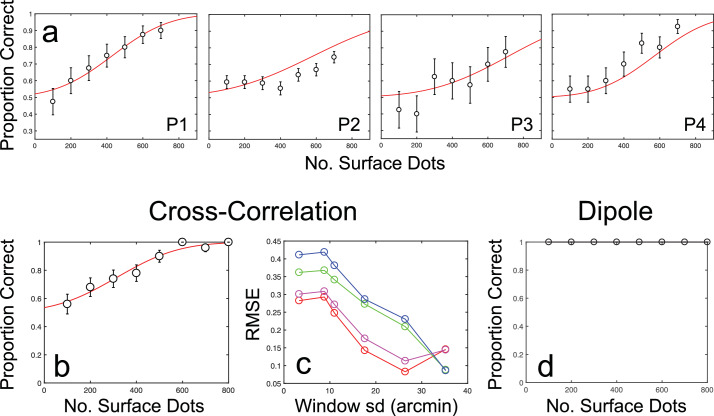
Results of Experiment 3. (a) Results for each participant (P1 is author RG), together with fitted scaled cumulative Gaussian functions. Error bars show binomial standard errors. (b) Example results for the cross-correlation model with a window standard deviation of 26.4 arcmin (close to the best-fitting window size; see Experiment 3 Results and discussion for details). (c) Prediction errors for the cross-correlation model, plotted as RMSE against window standard deviation for each participant. (d) Dipole model performance. As in Experiments 1 and 2, the dipole model performs at ceiling-level across all tested conditions.

Figure 6a shows the results for Experiment 3, plotted as proportion correct scores against the number of surface dot elements. For all observers, increasing the number of surface dots increased the ability to discriminate the direction of cyclopean orientation, despite a concomitant increase in the number of masking dots. Thresholds for 75% correct orientation discrimination averaged 573 dots across all four observers. Individual thresholds were 428, 583, 714, and 571 dots, with 95% CIs of ±6, ±211, ±76, and ±113 dots, respectively.

#### Modeling results

Modeling results for the manipulation of element number in Experiment 3 are shown in Figures 6b through 6d. Once again, the performance of the cross-correlation model is qualitatively similar to that of human observers (Figures 6b and 6c). As in Experiment 1, the match between human and cross-correlation model performance is best for larger window sizes, with a best-fitting window standard deviation of 31 arcmin (with 95% CIs of ±10.04 arcmin) (Figure 6c). At smaller window sizes, the cross-correlation model approaches ceiling performance even with smaller numbers of stimulus elements. The dipole model also maintains ceiling-level performance with the manipulation of element number (Figure 6d). As with Experiments 1 and 2, these results again suggest that antiphase masking effects primarily involve the impairment of measurement processes. Once again, however, the dependence on large correlation window sizes means that an explanation based on absolute disparity measurement alone runs contrary to expectations (Allenmark & Read, 2011; Nienborg et al., 2004) and requires one to hold that cyclopean surface perception is driven by highly suboptimal processes.

## Experiment 4: Random disparity masking of cyclopean orientation

### Stimuli

As in previous experiments, participants in Experiment 4 were presented with random-dot-defined depth sinusoids in the presence of binocular masking dots. Here, however, masking dots were assigned random disparities, drawn from a uniform distribution with range ±1.1 arcmin, centered at fixation. Given this random distribution of disparities, mask-to-surface dot ratios could be increased well beyond the maximum value used in Experiment 1. We presented participants with stimuli in which mask-to-surface dot ratios varied between 0.2 and 3.6 across seven uniformly spaced levels. The number of dots specifying the target surface was also varied. Three of the five participants were presented with target surfaces comprised of either 60, 100, or 200 dots, whereas the remaining participants were presented with target surfaces comprised of either 60, 80, 100, 150, or 200 dots. These were equivalent to target surface dot densities of between 3.1 and 10.3 dots per degree^2^. Mask-to-dot ratio thresholds were obtained for each target surface dot density.

### Results and discussion

#### Psychophysical results

**Figure 7.**
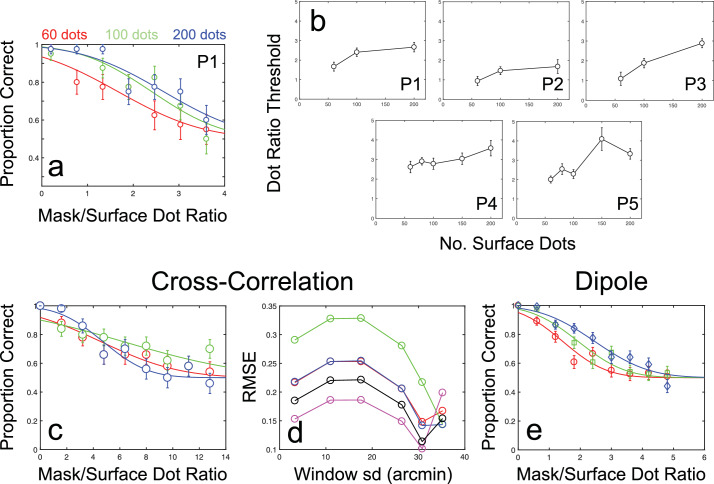
Psychophysical and modeling results for Experiment 4. (a) Results for an example participant (P1 is author RG), showing proportion correct scores against mask-to-surface dot ratios for three quantities of surface dot numbers. Error bars show binomial standard errors. (b) Threshold mask-to-surface dot ratios for each participant, as a function of the number of surface dots. Error bars show the standard deviation of best-fitting thresholds, obtained via bootstrapped resampling. (c) Example psychometric functions for the cross-correlation model at a window standard deviation of 30.8 arcmin (close to the best-fitting window size; see Experiment 4 Results and discussion for details). Error bars show binomial standard errors. (d) Prediction errors for the cross-correlation model, shown for each participant as RMSE against window standard deviation. (e) Results of the dipole model, plotted as proportion correct scores against mask-to-surface dot ratios for different number of surface dots. Error bars show binomial standard errors.

Figures 7a and 7b shows the results of Experiment 4, plotted as psychometric functions for an example participant, and as 75% correct mask-to-surface dot ratio thresholds for each participant, across different surface dot numbers. Mask-to-surface dot ratio thresholds increased slightly with increasing numbers of surface dots, from an average threshold ratio of 1.67 for 60 surface dots to an average threshold ratio of 2.83 for 200 surface dots (*F_2,8_* = 25.31, *p =* 0.0003 on a Repeated Measures ANOVA). Critically, however, the values of these threshold ratios provide an important comparison to results from Experiment 1. Although mask-to-surface dot ratios in Experiment 1 were necessarily limited by the antiphase structure of the masking stimulus, this was not the case for the randomly distributed masks used in Experiment 4. An increase in the ratio of mask-to-surface dots beyond a value of 1 continued to increase the effectiveness of the mask. This is critically important, as threshold ratios in Experiment 4 were much higher than those found with antiphase masks; observed threshold ratios fell between a minimum of 0.94 and a maximum of 4.1. The 95% CIs for these thresholds averaged ±0.61 across participants and conditions, although larger CIs were found for participants P4 and P5 in the 150 and 200 dot conditions, respectively. Here 95% CIs were ±1.1 and ±1.72 for surface-to-mask ratio thresholds of 3.58 and 4.1. These results suggest that randomly distributed noise masks are much less effective than the previously employed antiphase masks in disrupting cyclopean surface perception.

#### Modeling results

Model performance in Experiment 4 (Figures 7c–e) was markedly different to the previous experiments. Unlike antiphase masks, increases in mask-to-surface dot ratios for random masks had relatively little effect on orientation discrimination performance for the cross-correlation model. Threshold ratios of between 8 and 16 were found across multiple window sizes. Even at larger window standard deviations (i.e., 26.4 and 30.8 arcmin), in which human comparable performance had been found in Experiment 1, thresholds ratios were between 4.5 and 10. These thresholds far exceed the thresholds of between 1.62 and 3.56 seen in our participants. Beyond these window sizes, performance collapses almost completely, with proportion correct scores reaching only slightly above threshold level, even in the complete absence of any masking stimulus. Despite this collapse in performance, the best-fitting window standard deviation was found at this larger range. Best-fitting window standard deviation was 31.7 arcmin, with 95% CIs of ±7.33 arcmin. RMSE measures at this window standard deviation averaged 0.16 across observers. These results suggest that the measurement factors limiting performance with the antiphase mask are not the same as those limiting performance with the random mask.

Results for the dipole model in Experiment 4 are shown in Figure 7e. Although the cross-correlation model performed far in excess of human observers when presented with a randomly distributed mask, this was not the case for the dipole model. Dipole model thresholds were at ratios of between 1.4 and 2.5, broadly comparable to those found for human observers, and showed a clear increase with increasing numbers of surface dots. RMSE measures for the dipole model were substantially lower, at a value of 0.02, than for the best-fitting cross-correlation model, with a mean difference of 0.141. The 95% CIs of ±0.139 for the difference between model RMSEs indicate a significantly better fit for the dipole model than the best-fitting cross-correlation model, despite its lack of free parameters. This contrasts markedly with dipole model performance in Experiments 1 through 3, in which near ceiling-level performance was maintained throughout and fits to human data were poor. The improved fit and match to changing thresholds patterns for the dipole model suggest that the effects of the random disparity mask are better accounted for by complexity-limited processes that represent the relative disparity information available in the stimulus. We consider the possible nature of these processes and the limitations of our dipole approach later.

## Discussion

This article has demonstrated the effects of two types of cyclopean masks on the perception of disparity-defined form. The ability to discriminate the orientation of depth sinusoid stimuli was impaired by the presence of both an antiphase depth sinusoid mask and by a mask containing randomly distributed dots in depth. Comparison with the performance of two models of cyclopean surface perception suggests that masking effects occur at multiple levels of disparity processing, affecting both disparity measurement and the description of relative disparity structures. Results consistent with the effects of measurement noise accounted for performance with antiphase masks, although the scale of this measurement noise varied between experiments. Random disparity masks produced results consistent with the complexity-limited impairment of cyclopean linkage processes. We consider these differing effects in detail later.

### Measurement noise as a limiting factor in cyclopean perception

At the earliest stages of binocular integration, the brain measures disparities in an absolute (i.e., retinal) frame of reference (Cumming & Parker 1999). As noted earlier, these processes appear to be well-described by the disparity energy model (Fleet et al., 1996; Ohzawa, DeAngelis & Freeman, 1990; Read, 2005) and can be broadly understood as a process of binocular cross-correlation (Banks et al., 2004; Fleet et al., 1996; Goutcher & Hibbard, 2014). The acuity of binocular vision is limited by the resolution at which these measurement processes operate, with resolution dependent on the size of the cross-correlation window (Banks et al., 2004). Larger windows provide the measurement process with increased information but are likely to encounter differences in disparity across the windowed area, resulting in decreased signal-to-noise ratios. Thus the optimal window size for cross-correlation depends on the cyclopean frequencies present in the stimulus (Kanade & Okutomi, 1994).

Our results show that masking effects for the antiphase stimulus are consistent with effects of measurement noise arising with large cross-correlation windows. Critically, however, cross-correlation model performance impairments are found at highly suboptimal, very large, window sizes. This effect of larger window sizes is the result of an increase in the presence of information consistent with each disparity across the image and can be considered as comparable to the effects of increasing cyclopean frequency. To argue, however, that early measurement noise is the determining factor in such cyclopean masking effects is to suppose that the systems for measuring absolute disparity are poorly suited to the stimuli used in our experiments. This seems a spurious argument, given the sensitivity of disparity processing in a range of other tasks (e.g., Stevenson et al., 1989; Tyler & Kontsevich, 2001). In addition, these large window sizes were also at odds with previous estimates, defined in Equations 2 and 3 (Allenmark & Read, 2011; Nienborg et al., 2004; Smallman & MacLeod, 1994), and with estimates of the effects of correlation window size on the disparity gradient limit (Filippini & Banks, 2009). These previous estimates suggest window standard deviations of between approximately 4 and 12 arcmin in Experiments 1, 3, and 4, compared with the best-fitting window standard deviations of 24.5, 31, and 31.7 arcmin, respectively. Notably, in Experiment 2, in which previous suggestions for window size (Allenmark & Read, 2011; Nienborg et al., 2004) would align with larger correlation windows, our results are actually consistent with a smaller best-fitting cross-correlation window of standard deviation 15.5 arcmin.

One possible explanation is that window size is not the sole noise-limited factor for disparity measurement. For example, performance may be driven by processing inefficiency, or by suboptimal decision strategies. Although the decision rule for the cross-correlation model mirrors that used by other authors (e.g., Allenmark & Read, 2010, Allenmark & Read, 2011) it should only be considered as an approach appropriate to the experimental task. It cannot reflect the wider decision strategies employed by the visual system. In addition, later processing stages could add further noise or distortion to disparity measurement processes. These could involve both additional absolute disparity processes, such as disparity averaging (e.g., Cammack & Harris, 2016) or processing at the relative disparity level (e.g., Goutcher et al., 2018). Thus the apparent limiting effects of large cross-correlation windows may reflect the scale over which higher resolution absolute disparity measurements (i.e., those arising from smaller cross-correlation windows) are brought together to feed into subsequent mechanisms for the measurement of relative disparity. Note that this potential spatial scale effect of relative disparity processing is distinct from the point-to-point measurements of relative disparity in the dipole model. Instead, such effects could reflect the smoothing of absolute disparities seen with the use of cyclopean frequency-tuned hypercyclopean filters (Goutcher et al., 2018; Hibbard, 2005; Serrano-Pedraza & Read, 2010; Tyler, 2012; Tyler & Kontsevich, 2001).

It is also unlikely that any noise in relative disparity or hypercyclopean processing reflects a preponderance of relative disparity signals to which the visual system is not well-tuned. For example, one may predict poor measurements of relative disparity for stimuli containing large disparity gradients (i.e., for stimuli in which large disparity differences occur over a small local area). This is, however, not the case for the stimuli in our experiments. Approximately 90% of stimulus dipoles have disparity gradients of <0.5 for random masks at a mask-to-surface dot ratio of 4.8, while 90% of dipoles have gradients of <0.6 for antiphase masking stimuli at the limiting mask-to-surface dot ratio of 1. Thus even for high noise stimuli, the majority of dipoles fall well below the gradient limit of 1 for binocular fusion (Burt & Julesz, 1980) and the limit of 3 for stereoscopic depth perception and surface segmentation (McKee & Verghese, 2002).

Although the antiphase mask effects in Experiments 1 through 3 are consistent with measurement noise-related processing limitations, the results of Experiment 4 are not. Thresholds for the random disparity mask in Experiment 4 were far in excess of human performance across a wide range of correlation window sizes and failed to show an increase with surface dot density. This better-than-human performance was even observed for the larger, highly suboptimal, window sizes in which cross-correlation performance best matched human performance in Experiments 1 and 3. These results suggest that factors other than measurement noise are critical in accounting for the effects of the random disparity mask.

### Dipole distributions as a “minimal linkage” description of disparity differences

In addition to examining the performance of the cross-correlation model, this article also introduced a dipole model for the consideration of relative disparity stimulus content. As noted earlier, this model should not be considered as an account of the processes involved in relative disparity measurement (e.g., Assee & Qian, 2007; Hibbard & Goutcher, 2016; Thomas et al., 2002; Zhaoping, 2002) or of the description of cyclopean structures using such measurements (e.g., Tyler, 2012). Instead the model provides a summary description of one “*minimal* *linkage*” measure of relative disparity content and allows for a judgement of whether such a description can, in principle, support cyclopean orientation discrimination. Thus using this model, we may conclude that the orientation of the family of stimuli used in Experiments 1 through 3 can be discriminated on the basis of dipole distributions alone, without any consideration of positional or absolute disparity information. Comparisons of results between the dipole and cross-correlation models thus allow us to distinguish between measurement noise-limited and complexity-limited cyclopean perception.

For the random disparity mask used in Experiment 4, the dipole model shows performance limitations similar to human behavior. As with humans, and unlike the cross-correlation model, the dipole model shows an increase in mask-to-surface dot ratio thresholds with increasing surface dot density. In tandem with the discrepancies between human and cross-correlation model performance, this suggests that performance limitations with random disparity masks involve the complexity-limited description of relative disparity information, rather than a noise-based impairment of absolute disparity measurements. Thus random disparity masks primarily affect performance by increasing stimulus complexity, rather than increasing measurement noise.

As with the cross-correlation model, although the decision stage of the dipole model is ideally suited to the experimental task, it cannot be considered as a plausible account of the visual system's processes for interpreting relative disparity measurements. In particular, to allow for the proper encoding of surface structure, such processes most likely involve the further comparison of relative disparity measurements over space. Such processes would require the positional information already absent from the distributions of the dipole model. Use of such positional information, for the further grouping of relative disparity measurements across the image, could help to improve dipole model performance, allowing it to match the full range of observed human performance even in the presence of measurement noise and/or processing inefficiency.

### Describing cyclopean structures

Comparison of modeling results to human performance suggests that cyclopean masking effects arise at the level of both absolute disparity and relative disparity processing. Antiphase masking effects are consistent with the effects of measurement noise, whereas random disparity masks affect performance in a manner consistent with the disruption of relative disparity information. Note that impairments in dipole model performance are not owing to errors or uncertainty in relative disparity measurements but owing to the impact of mask-related dipoles on the dipole distribution. These reductions thus represent a masking of task-relevant relative disparity information: mask-related dipoles hide the stimulus structure without impairing the initial measurements on which such structure depends. As such, they illustrate a limitation in the visual system's ability to make effective use of summary descriptions of stimulus content. Limitations in the use of such summary descriptions are also evident in the perception of stereoscopic volume stimuli (Goutcher & Wilcox, 2016). Understanding the descriptions of disparity content available to the visual system will be a critical step in understanding how disparity measurement processes underpin the perception of cyclopean form.

Studies of the mechanisms responsible for the perception of cyclopean structure have revealed multiple processes, involving the action of multiple neural sites (Neri, 2004; Parker, 2007). Although there is evidence for a progression from absolute disparity selectivity in primary visual cortex to relative disparity selectivity in areas V2 and V4 (Fang et al., 2018; Thomas et al., 2002; Umeda et al., 2007), there is little consensus on either the nature of the transformations involved in this progression, or their computational purpose (cf., Assee & Qian, 2007; Hibbard & Goutcher, 2016; Zhaoping, 2002). Tyler (1975, 2012) has suggested a progression from initial disparity measurement units to units with frequency-tuned selectivity for depth sinusoids. The tuning properties of these “hypercyclopean” channels have been used to account for orientation dependencies in sensitivity to cyclopean form (Hibbard, 2005; Serrano-Pedraza & Read, 2010; Tyler & Kontsevich, 2001) and structure-related biases in perceived depth (Goutcher et al., 2018). Such mechanisms would encode much of the information contained within the dipole distributions examined here, although also subject to those aspects of measurement noise on which antiphase masking effects appear to depend. As discussed earlier, the effects of hypercyclopean processing may also provide a more biologically plausible basis for the cross-correlation model's dependence on large correlation windows for matching human performance.

### Conclusion

The experiments reported in this article demonstrate structure-dependent effects of cyclopean masking stimuli on the perception of disparity-defined form. Through comparison with two different modeling approaches, these effects are shown to be consistent with differing processing limitations. Although the effects of antiphase masks are consistent with processing limitations at the level of measurement noise, random disparity masks appear to impose a complexity-based limitation on performance, related to the description of the relative disparity content of the stimulus. Further use of these different mask types should prove useful in determining the mechanisms underlying these different aspects of cyclopean form perception.

## Supplementary Material

Supplement 1
