# Supplementary figures and images for: Impairment of cyclopean surface processing by disparity-defined masking stimuli
Source: J Vis. 2020 Feb 10;20(2):1. doi: 10.1167/jov.20.2.1 (PMC7331773; doi:10.1167/jov.20.2.1)

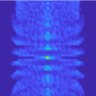

Supplement: Supplement 1 [file jovi-20-2-1_s001.jpg]
